# Molecular classification and immunotherapy in fertility-sparing treatment for endometrial cancer: opportunities and challenges

**DOI:** 10.3389/fonc.2026.1853596

**Published:** 2026-07-09

**Authors:** Jing Li, Shixiang Dong, Dongdong Hu, Wen Feng

**Affiliations:** 1 Department of Gynecology, The First People’s Hospital of Lianyungang, Lianyungang, China; 2 Department of Gynecology, The First People’s Hospital of Lianyungang, Affiliated to Kangda College of Nanjing Medical University, Lianyungang, China; 3 Department of Gynecology, Lianyungang Hospital Affiliated to Xuzhou Medical University, Lianyungang, China

**Keywords:** endometrial cancer, fertility spare, immune checkpoint inhibitors, mismatch repair deficiency, molecular classification

## Abstract

Endometrial cancer (EC) is one of the most common malignant tumors in women, and its incidence is rising continuously worldwide. According to recent global cancer statistics, there were about 417,000 new cases and 97,000 deaths from EC in 2020(1). The standard treatment for EC is hysterectomy, which undoubtedly deprives some young patients of their fertility. Fertility-sparing treatment (FST) for endometrial cancer allows these women to preserve fertility before undergoing standard treatment. Traditional fertility-sparing regimens are mainly based on progestin. However, in recent years, with the application of molecular classification, some studies have found that among patients undergoing FST, women with POLE mutations (POLE-mut) often have a higher complete remission (CR) rate, while those with P53 abnormal (P53abn) or mismatch repair deficiency (dMMR) have poor prognosis. Among them, patients with dMMR endometrial cancer seem to have a higher recurrence rate after achieving complete remission through progestin treatment. Currently, fertility-sparing treatment regimens for dMMR patients are being gradually studied, and immune checkpoint inhibitors (ICIs) may play a key role in the treatment.

## 1 Introduction

Endometrial cancer is one of the most common malignant tumors of the female reproductive system. Data from the SEER website show an incidence rate of 4.5 per 100,000 in individuals under 40 years of age (1). The increasing incidence of endometrial cancer in young women may be related to factors such as obesity and delayed childbearing (2). The standard treatment for endometrial cancer is total hysterectomy, which results in loss of fertility for women who wish to preserve childbearing potential (3, 4). However, with the deepening of TCGA molecular classification research, molecular classification has provided new evaluation and treatment strategies for young women seeking fertility-sparing treatment for endometrial cancer. Patients with different molecular subtypes have significantly different prognoses. Among them, POLE-mut has the best prognosis, dMMR and P53 wild-type (P53wt) have intermediate prognosis, and P53abn has the worst prognosis. As a distinct molecular subtype, dMMR subtype often indicates a poor prognosis as well as insensitivity to radiotherapy and chemotherapy (2–6). However, studies have found that immune checkpoint inhibitors(ICIs) show promising therapeutic effect on advanced/recurrent endometrial cancer (7–9). In some studies on fertility preservation, it has been gradually observed that anti-programmed cell death protein 1(PD-1) inhibitors may have a significant efficacy in fertility-sparing treatment of endometrial cancer (10, 11). This review aims to analyze the role of molecular subtypes in endometrial cancer FST and explore the prospect of ICIs in FST.

## 2 Traditional fertility-sparing treatment regimens for endometrial cancer

### 2.1 Criteria for endometrial cancer FST

Based on the ESGO/ESHRE/ESGE guidelines, the core eligibility criteria for fertility-sparing treatment (FST) of endometrial cancer can be summarized as follows:1) Well-differentiated endometrioid adenocarcinoma, grade 1, FIGO stage IA, without myometrial invasion; 2) Absence of contraindications to progestin treatment or pregnancy; 3) Strong desire to preserve fertility; 5) No evidence of suspicious metastatic lesions on imaging; 6) Provision of fully informed consent regarding the risks associated with fertility-sparing treatment under multidisciplinary team (MDT) guidance. For grade 2 tumors or those with minimal myometrial invasion (1–2 mm), FST may be considered on a case−by−case basis, as evidence is limited but promising. For these patients, hysteroscopic tumor resection followed by progestin therapy is an optional treatment regimen. In patients with Lynch syndrome, FST should also be discussed individually, with clear counseling about the higher risk of persistence or recurrence compared with other patients, and the need to rule out synchronous cancers (especially ovarian cancer). In contrast, patients with the P53abn are not eligible for any form of fertility−sparing therapy due to its aggressive behavior and extremely poor prognosis (12).

### 2.2 Hysteroscopy

Before initiating FST, patients should be fully informed that FST is not the primary treatment of choice, and that total hysterectomy must be performed promptly in the event of treatment failure or after childbearing is completed (13). Current studies suggest that hysteroscopic resection combined with progestin-based therapy is the most effective treatment method. In the past, dilatation and curettage (D&C) was the standard method for obtaining pathological specimens of endometrial cancer, however, it is associated with a considerable false-negative rate. Currently, more studies recommend the use of visual specimen collection methods, such as hysteroscopic surgery. Recent studies indicate that hysteroscopy is more accurate than D&C (14–16). Pathological diagnosis is obtained through hysteroscope to determine whether the patient meets the criteria for fertility-sparing treatment. Mazzon et al. first described a three−step hysteroscopic resection of focal endometrioid endometrial carcinoma, consisting of resection of the tumor lesion, the adjacent endometrium (4–5 mm), and the underlying myometrium (3–4 mm). Following pathological confirmation of grade 1 disease without myometrial invasion, megestrol acetate (160 mg daily) was administered for six months. This combined approach achieves complete remission rates of 90%–95.3%, significantly higher than progestin monotherapy (76.3%–77.7%), and is also associated with higher live birth rates (17).

### 2.3 Progestin-based therapy

Conventionally, progestin-based agents have been the mainstay of treatment. Medroxyprogesterone acetate (MPA) and megestrol acetate (MA) are the most commonly used progestins. Compared with MPA and other hormonal agents, MA is associated with higher remission rate, which may be attributed to its relatively higher oral bioavailability (12, 18). However, dosages vary across studies and may affect efficacy. Systematic reviews have demonstrated that high-dose progestin regimens achieve superior outcomes, the recommended daily doses are 160–320 mg for MA and 400–600 mg for MPA (13). The 2024 National Comprehensive Cancer Network(NCCN) guidelines recommend LNG-IUS as first−line FST; other progestins (MPA, MA) are listed as alternatives. Combination therapy with LNG-IUS plus oral progestins or GnRH agonists(GnRH-a) achieves favorable remission and low recurrence rates (18). A meta-analysis by Suzuki et al. evaluated the complete remission (CR) rate within one year of treatment using oral progestins or LNG-IUS for fertility-sparing treatment in patients with endometrial cancer. The study found that LNG-IUS was associated with a higher optimal combined CR rate (86% vs 66%) and a higher combined pregnancy rate (58% vs 44%) compared with oral progestins in the fertility-sparing treatment of early-stage endometrial cancer (19). However, to date, only observational studies are available, and well-designed randomized controlled trials (RCTs) directly comparing the efficacy and safety of LNG-IUS versus oral progestins remain lacking (20, 21). Therefore, treatment selection in clinical practice should be individualized based on patient-specific factors. Various other regimens have been described in the literature, including hydroxyprogesterone caproate, norethisterone acetate, natural progesterone, aromatase inhibitors (letrozole, anastrozole), and combined oral contraceptives. However, comparative studies are insufficient to confirm their relative efficacy (22, 23).

### 2.4 Follow−up and Management

Currently, no consensus exists regarding the optimal treatment duration. Earlier literature recommended discontinuing progestin therapy if no response was observed by six months (24, 25). However, a recent study demonstrated that 50% of women require more than nine months to achieve complete remission (CR) (26). Simpson et al. suggested that treatment duration should not exceed one year; beyond this point, dose adjustment or hysterectomy should be considered (27). During treatment, hysteroscopic assessment is recommended every three to six months. For patients with disease progression after six to nine months of progestin therapy, pelvic MRI is advised (28). Indications for hysterectomy include non−response to treatment (after 6–12 months), disease progression, recurrence, completion of childbearing, or inability to conceive (29). Oophorectomy should be decided on a case−by−case basis; if the ovaries are preserved, concomitant salpingectomy is recommended (30). For patients with a strong desire to preserve fertility who experience recurrence, a second conservative treatment may be considered on a case−by−case basis, although the evidence is limited (17).

Positive estrogen receptor (ER) and progesterone receptor (PR) expression is associated with a more favorable response to progestin−based conservative treatment and better prognosis. However, the accuracy of ER/PR as predictive markers is insufficient for routine clinical decision−making (31, 32). The ESGO guidelines state that ER/PR expression may be useful for patient counseling but that negative expression is not a contraindication for fertility−sparing treatment (12).

Assisted Reproductive Technology (ART) is recommended to improve pregnancy rates and shorten the time to conception after CR, without increasing the risk of recurrence. For young patients with good reproductive potential (e.g., age <35 years and no infertility history), natural conception may be attempted within a defined timeframe of 6–9 months under close surveillance. Ovarian stimulation protocols should be individualized, and the use of letrozole combined with gonadotropins is suggested based on evidence from breast cancer patients. ART decisions should be made by a multidisciplinary team including gynecologic oncologists and reproductive specialists. Early referral to fertility specialists is strongly encouraged to avoid unnecessary delays in achieving pregnancy.

### 2.5 Pregnancy outcomes

In a meta-analysis by Suzuki et al., the pooled pregnancy rate was 58% (95% CI 37-76) for oral progestin and 44% (95% CI 6-90) for LNG-IUD, with corresponding live birth rates of 39% and 24%, respectively. However, a single−center retrospective study by Simpson et al. reported a much lower live birth rate of only 7% overall (18% among those receiving ART), reflecting the inherent subfertility of this population. For the MSI−H/MMRd subtype, a systematic review by Zhang et al. found that spontaneous pregnancy after CR was rarely successful, whereas one study that actively utilized ART reported a 1−year cumulative pregnancy rate exceeding 60%. These findings underscore the importance of early referral to reproductive specialists and the urgent need for standardized reporting of fertility outcomes in future studies.

## 3 The guiding role of molecular classification in fertility-sparing treatment

### 3.1 Molecular classification

In 2013, The Cancer Genome Atlas (TCGA) proposed four molecular subtypes of endometrial cancer: POLE ultramutated, microsatellite instability-high (MSI-H), copy-number high (CN-H), and copy-number low (CN-L). To facilitate clinical application, the TCGA classification was refined into the TransPORTEC and ProMisE classification systems, which integrate POLE mutation status, mismatch repair protein deficiency, and P53 protein expression. The ProMisE classification categorizes endometrial cancer into four subtypes: POLE exonuclease domain-mutated type, mismatch repair deficiency type (MMRd, equivalent to MSI-H type), P53 wild-type(P53wt, equivalent to non-specific molecular profile), and P53abn type (equivalent to CN-H type) (1, 20). For clarity, this review will uniformly adopt ProMisE classification.

### 3.2 POLE-mut

In TCGA molecular typing, patients with POLE-mut account for only 7%, but have the best prognosis after standardized treatment. They are often younger and have a normal body mass index(BMI) (33). These patients represent a potential population for treatment de-escalation, i.e., reducing the extent of surgery while omitting radiotherapy or chemotherapy (34–36). Mutations in the exonuclease domain of POLE lead to the loss of proofreading function, resulting in the accumulation of mutations. This process contributes to the occurrence and development of various tumors. Pathogenic POLE mutations are closely associated with high tumor mutational burden, significant CD8+ cytotoxic T lymphocyte infiltration, and increased expression of immune checkpoint-related proteins such as PD-1 (37). Patients with POLE mutations account for a small proportion of those undergoing fertility-sparing treatment. Consequently, few studies have focused on fertility-sparing treatment in this subgroup, and the sample size of existing studies are limited. Most patients with POLE mutation have high-grade endometrial cancer, which is a contraindication for fertility-sparing treatment. Moreover, limited studies suggest that these patients may be insensitive to progestin therapy. In the study by Wang et al., POLE-mut was identified as an unfavorable factor for FST in patients with EC or EAH. Although the CR rate in POLE-mut patients was high (90.9%), the recurrence rate after CR reached 40%, which was substantially higher than that of the non-specific molecular profile (NSMP) group (21.7%). Moreover, two of the 11 POLE-mut patients (18.2%) developed ovarian cancer during FST, further compromising fertility outcomes. Relapse−free survival also showed a trend toward being lower in the POLE-mut subgroup compared with NSMP (p = 0.069). These findings suggest that despite the excellent postoperative prognosis typically associated with POLE-mut tumors, their behavior in the fertility−sparing setting is less favorable, warranting closer surveillance and individualized management (10). While Agustí et al. (2024) stated that POLE−mut tumors have an excellent prognosis in the definitive surgery setting. However, they noted that evidence for FST is very limited, based on very small sample sizes (ranging from 1 to 4 patients). They also highlighted that most POLE−mut tumors present with high−grade histology, which is traditionally a contraindication for FST, and therefore the safety of conservative management for this subgroup requires further investigation (38). Given the excellent prognosis of patients with POLE-mut, should the indications for conservative treatment be expanded to include stage IA disease with minimal myometrial invasion? Furthermore, can immunotherapy play a role in the fertility-sparing for these patients (39–41)?

### 3.3 P53abn

The P53abn subtype is generally considered to have the worst prognosis. Such mutations are detected in more than 90% of serous carcinomas, most mixed carcinomas, and some high-grade endometrioid carcinomas. It is usually associated with a greater invasiveness and a higher recurrence rate. Patients with this subtype have a poor response to traditional treatment, also, ER and PR positivity rates are significantly lower than those in other endometrial cancer subtypes (2, 5). Many literatures do not recommend fertility-sparing treatment for patients with P53abn. In a retrospective study conducted by Xu et al., a total of 90 patients who received FST were included, among whom one was classified as the P53abn type. This patient still did not achieve CR after 90 weeks of treatment. Peng et al. performed immunohistochemical analysis of MMR and P53 protein on endometrial specimens from 51 patients with endometrial atypical hyperplasia (EAH) who received fertility-sparing treatment. They evaluated response, recurrence, and progression rates based on dMMR, P53abn, and other baseline characteristics. Of these, 21.6% had MMR deficiency, and 11.8% had P53abn. The study found that EAH patients with dMMR and P53abn had a significantly increased risk of disease recurrence and progression (42).

### 3.4 dMMR

Patients with dMMR subtype account for approximately 20-30% of cases in TCGA molecular classification (33). This subtype is characterized by high microsatellite instability (MSI-H). Somatic mutations in DNA mismatch repair genes increase the risk of tumors in the uterus, ovaries, gastrointestinal tract, and other organs. This condition also leads to poor response to progestin therapy in the context of fertility-sparing treatment. Patients with dMMR undergoing fertility−sparing treatment have lower complete remission rates (approximately 62%) and significantly higher recurrence risks (approximately 41%). Chung et al. evaluated 27 endometrial biopsy samples before fertility-sparing treatment and performed molecular typing. Among the corresponding patients, 9 had mismatch repair deficiency. Their CR rate was significantly lower than that of patients with P53 wild-type (44.4%; 95% confidence interval 4.0%–85.0% vs. 82.2%; 95% confidence interval 71.0%–94.0%, P = 0.018) (40). Puechl et al. evaluated 58 patients with EC or EAH who received fertility-sparing treatment with intrauterine device (IUD). Among them, 37.9% had EC and 62.1% had EAH. The cohort included 44 patients with P53 wild-type, 6 with dMMR, 4 with P53abn, and 4 with POLE-mut. In this study, patients with P53abn had the shortest time to progression or definitive treatment, at 5.7 months (range: 4.6–6.8 months). Patients with POLE−mut and dMMR had the longest time to progression or definitive treatment (21.4 months and 20.9 months, respectively) (43). In a systematic review by Zhang et al. on the oncological and reproductive outcomes of fertility-sparing treatment in patients with MSI-H/dMMR endometrial cancer or EAH, ten retrospective studies were analyzed. The overall remission rate in this population was 61.8%, which was lower than that reported in the general population undergoing fertility-sparing treatment, while the recurrence rate was higher. The authors suggest that caution should be exercised when considering fertility-sparing treatment for patients with MSI-H/dMMR (44).

Defective MMR proteins are a hallmark of Lynch syndrome, an inherited disease. All endometrial cancer patients with dMMR should undergo Lynch syndrome evaluation, including MMR immunohistochemistry followed by MLH1 methylation testing (if MLH1−deficient) and germline genetic testing as indicated. Lynch syndrome accounts for approximately 3% of all ECs but up to 9% of those diagnosed under age 50. In Lynch syndrome patients undergoing FST, the complete remission rate is 75% but the recurrence rate is as high as 77.8%, significantly higher than that of sporadic dMMR or other molecular subtypes. The ESGO guidelines recommend that Lynch syndrome patients be counseled about the higher risk of persistence/recurrence and the need to rule out synchronous cancers (especially ovarian cancer). Fertility−sparing treatment in LS patients should be discussed on a case−by−case basis, and genetic counseling is mandatory (12, 40, 44–46).

### 3.5 P53wt

The P53wt accounts for approximately 50%-55% of endometrial cancer patients. These patients lack specific molecular characteristics, such as MMR protein deficiency, P53abn, and POLE-mut (38). Diagnosis of this subtype is based on the exclusion of the above three molecular characteristics rather than the confirmation of a specific biomarker. The overall prognosis of P53wt among the four molecular subtypes is intermediate (1, 2). Over a 10-year period, Ran et al. performed TCGA molecular classification on endometrial specimens from patients with endometrial cancer who received fertility-sparing treatment at their institution. They then integrated their findings with the results of three previous related studies. The study did not find statistically significant differences in CR rate and recurrence rate among patients with four different subtypes. P53wt appeared to have a similar prognostic risk to the MMRd subtype, with a recurrence rate of 29.9%. Among patients who received FST, 77 were classified as non-specific molecular profile (NSMP). The estimated recurrence rate was 30%, and 13 patients (16.9%) underwent hysterectomy (40). Dagher et al. recently reported a study of 20 young patients who received FST. In their cohort, NSMP tumors had the best response to progesterone treatment, with 63% achieving complete remission (32).

CTNNB1 (β−catenin) mutation is frequently found in the P53wt molecular subtype, particularly in low−grade, early−stage endometrioid tumors. The role of CTNNB1 mutation in fertility−sparing treatment remains controversial. Some studies suggest that CTNNB1 mutation is associated with a higher risk of recurrence in low−grade, early−stage endometrial cancer, while others have not confirmed this association (47, 48). The ESGO guidelines indicate that mutational analysis of CTNNB1 and P53 may help identify a subset of patients with low−grade, early−stage endometrial carcinoma who are at higher risk of recurrence (12). Therefore, CTNNB1 mutation testing may be considered for risk stratification in P53 patients undergoing fertility−sparing treatment, but further prospective studies are needed to validate its predictive value.

Currently, most studies supporting the use of molecular classification to predict outcomes of FST are small and retrospective, with heterogeneous conclusions. The overall evidence base remains weak, and molecular classification is not yet sufficiently robust to serve as a rigid criterion for clinical decision-making in FST. The study by Ran et al. suggested that molecular classification has no predictive significance for the prognosis of fertility-sparing treatment. However, these findings were inconsistent with the results of the other three studies included in their analysis (49). While the study by Chung et al. indicated that molecular classification has prognostic significance in fertility-sparing treatment, and that MMR status can serve as a prognostic predictor (40). Falcone et al. believe that although the sample sizes of existing studies are limited, the available data suggest promising results (50). Puechl et al. analyzed 58 patients with EC or EAH patients treated with LNG-IUS and found that patients with P53abn had the worst prognosis, suggesting that molecular classification can help predict tumor progression (43).Although current data do not support routine clinical use of molecular classification in FST, the ESGO guidelines recommend molecular profiling for all young patients with grade 1, low-stage endometrial cancer who desire fertility preservation. IHC identifies MMRd tumors and detects patients at high risk for Lynch syndrome. If confirmed, patients should receive cancer risk counseling. When FST is contemplated, P53 mutation status must be assessed by molecular testing. Patients with p53abn should not receive conservative therapy owing to their very poor prognosis (12).

## 4 Overview of immune checkpoint inhibitors in the treatment of endometrial cancer

In 2002, the concept of “cancer immunoediting” was proposed, indicating that the immune system can both hinder and promote tomor growth. In the endometrium, epithelial cells act as a physical barrier, while the immune system contributes to the production of defensins and other immune mediators, as well as antigen presentation. Under normal circumstances, PD-1 inhibits autoimmunity, limits infection-induced damage to healthy tissues, and promotes self-tolerance. Cancer immunoediting is divided into three stages: elimination, equilibrium, and escape. Following the elimination stage, residual tomor cells may enter a dormant state and reach equilibrium. If these cells succeed in establishing an immunosuppressive microenvironment, they transition to the escape stage. T cells and other immune cells express key components of immune checkpoint signalling, such as PD-1, programmed death-ligand 1(PD-L1), and cytotoxic T-lymphocyte-associated protein 4 (CTLA-4). Immune checkpoints contribute to immune escape by inhibiting activated T cells, thereby preventing tomor cells from being eliminated by the immune system. The PD-1/PD-L1 axis triggers the recruitment of tyrosine phosphatase Src homology 2 domain-containing protein tyrosine phosphatase 2 (SHP2), which dephosphorylates proximal signalling regions of the T cell receptor. This dephosphorylation leads to a negative co-stimulatory effect, inhibiting T cell activation and enabling immune evasion (7, 8, 51, 52).

**Figure 1:**
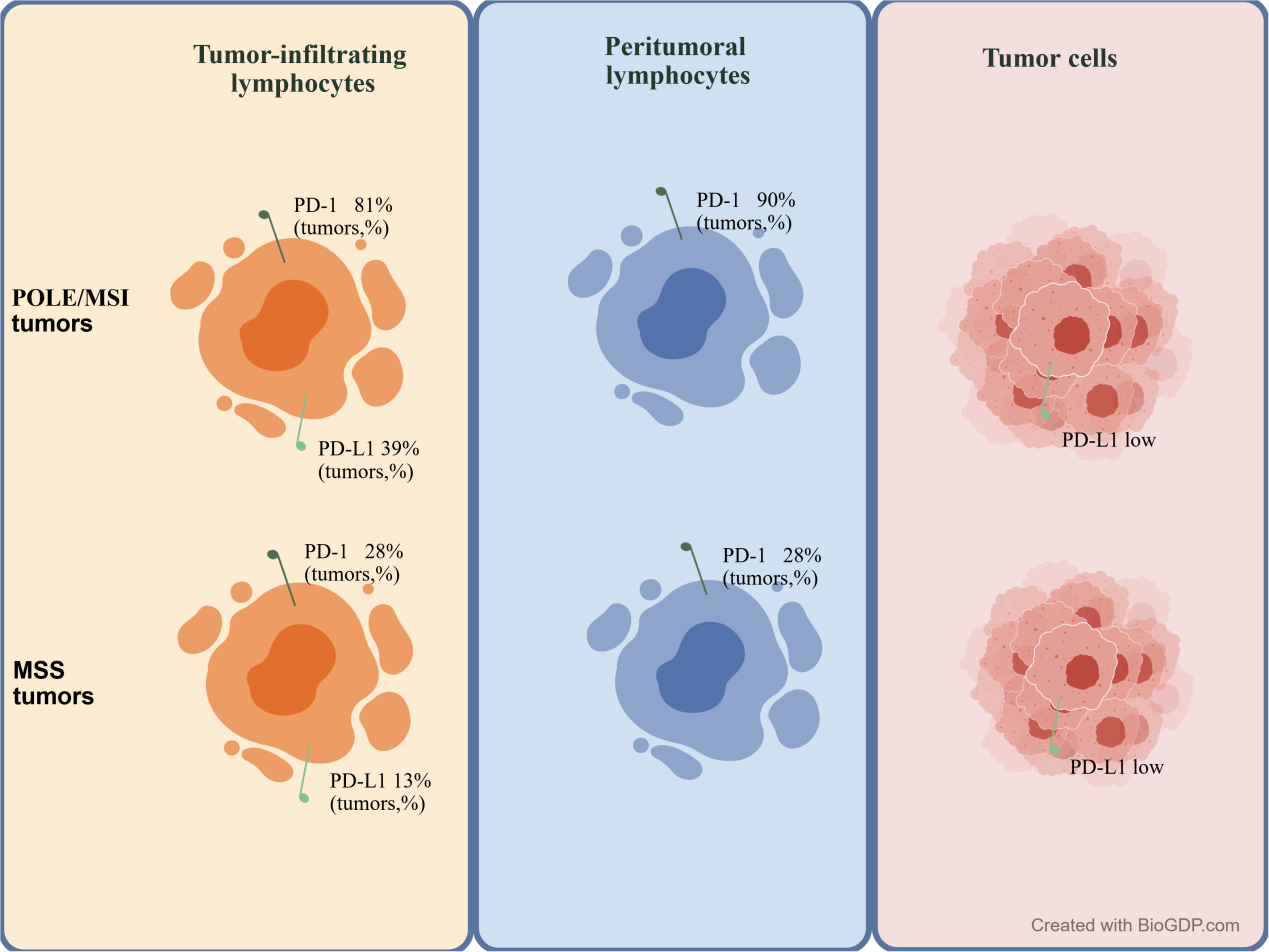
Schematic comparison of PD-1 and PD-L1 expression between POLE/MSI and MSS endometrial cancers based on the findings of Howitt et al. (2015). According to their study, PD-1 is highly expressed on tumor-infiltrating lymphocytes (TILs) and peritumoral lymphocytes in POLE/MSI tumors (81% and 90%, respectively), and PD-L1 is frequently expressed on intraepithelial immune cells (39%), whereas PD-L1 expression on tumor cells is low. In contrast, MSS tumors show low PD-1 expression (28% on both TILs and peritumoral lymphocytes) and low PD-L1 expression on intraepithelial immune cells (13%). This figure was created Created with (54).

Both PD-1 and PD-L1 inhibitors are highly expressed in EC cells. PD-1 is expressed in approximately 60%-65% of cases, while PD-L1 expression ranges from 25% to 70% (7, 9). In the study by Howitt et al., it was found that in POLE−mutated and MSI endometrial cancers, PD−L1 expression is significantly more frequent in intraepithelial immune cells compared with MSS tumors (39% vs 13%; P = 0.02), whereas PD−L1 expression in tumor cells is rare. This finding is clinically relevant because response to anti−PD−L1 therapy has been shown to correlate with PD−L1 expression in tumor−infiltrating immune cells rather than in tumor cells. Together with the high neoantigen loads, abundant CD3+/CD8+ tumor-infiltrating lymphocytes (TILs), and high PD−1 expression in TILs and peritumoral lymphocytes (81−90%), these observations support that POLE−mutated and MSI endometrial cancers are excellent candidates for PD−1/PD−L1−targeted immunotherapies (53). A schematic diagram summarizing the differences in PD−1 and PD−L1 expression between POLE/MSI and microsatellite stable (MSS) tumors is presented in **Figure 1**.

Numerous studies have demonstrated the efficacy of ICIs in recurrent and advanced EC. Multiple RCTs investigating ICIs for advanced/recurrent EC have been published and subsequently received Food and Drug Administration (FDA) approval. The RUBY trial was the first clinical trial to demonstrate statistically significant and clinically meaningful improvement in OS with the combination of immunotherapy and chemotherapy in patients with primary advanced or recurrent EC. Based on this phase III study, the NCCN guidelines list carboplatin/paclitaxel/dostarlimab as the preferred primary or adjuvant treatment regimen for stage III–IV endometrial cancer. On July 31, 2023, the FDA approved dostarlimab in combination with carboplatin and paclitaxel, followed by single-agent dostarlimab, initially for patients with dMMR primary advanced or recurrent EC. On August 1, 2024, the indication was further expanded to include all patients with primary advanced or recurrent EC, regardless of MMR or MSS status. The DUO−E/GOG−3041/ENGOT−EN10 trial investigated whether the addition of the anti−PD−L1 antibody durvalumab to carboplatin and paclitaxel could improve outcomes in patients with advanced or recurrent EC. In 2024, the FDA approved durvalumab plus carboplatin and paclitaxel, followed by single-agent durvalumab, for adult patients with dMMR primary advanced or recurrent endometrial cancer. Of note, the phase 3 DUO-E/GOG-3041/ENGOT-EN10 trial demonstrated that durvalumab plus carboplatin/paclitaxel followed by maintenance durvalumab, with or without olaparib, yielded a statistically significant and clinically meaningful progression-free survival (PFS) benefit in patients with advanced or recurrent endometrial cancer. Compared with the control arm, the durvalumab plus olaparib arm reduced the average risk by 45%, and the durvalumab monotherapy arm reduced the average risk by 29%. This represents the first evidence that adding a PARP inhibitor to a therapeutic regimen provides incremental benefit (55). These findings establish a new treatment paradigm for advanced or recurrent endometrial cancer, integrating immunotherapy with PARP inhibition. Preplanned exploratory analyses comparing durvalumab plus olaparib with durvalumab monotherapy showed that olaparib’s benefit was mainly observed in the proficient mismatch repair(pMMR) subgroup. By PD−L1 status, a clinically meaningful PFS benefit was seen in the PD−L1−positive subgroup, whereas the benefit was more modest in the PD−L1−negative subgroup. NRG−GY018, a double−blind, placebo−controlled, randomized phase III trial, evaluated pembrolizumab added to chemotherapy in advanced or recurrent endometrial cancer (excluding carcinosarcoma). Subgroup analyses of primary and secondary endpoints favoured the pembrolizumab arm in both dMMR and pMMR cohorts, irrespective of MMR status. Based on these findings, the FDA approved carboplatin/paclitaxel/pembrolizumab as a first−line preferred regimen for stage III–IV endometrial cancer in 2024. In the phase 3 KEYNOTE-B21 trial, the addition of pembrolizumab to chemotherapy was further validated to improve disease-free survival (DFS) in patients with dMMR endometrial cancer. The 2025 NCCN guidelines recommend this regimen for patients with stage III–IV dMMR endometrial cancer.

Given compelling results from high−quality studies of ICIs in advanced or recurrent endometrial cancer—particularly dMMR tomors—some investigators have begun exploring ICIs in the fertility−sparing therapy (FST) setting. In a retrospective cohort study, Wang et al. evaluated the outcomes of fertility-sparing treatment in 118 patients with EC/EAH who received fertility-sparing treatment at their centre over a 3-year period. Among these patients, 11 were classified as dMMR, and 6 achieved complete remission. Of these 6 patients, one received progestin monotherapy; three were insensitive to progestin and subsequently switched to combination therapy with progestin and PD-1 inhibitor; and two received combination therapy with progestin and PD-1 inhibitor at initial treatment. The researchers suggest that immunotherapy may be an effective fertility-sparing option for patients with dMMR. This retrospective study has several limitations, including the inclusion of only eight patients with endometrial atypical hyperplasia (EAH) and the use of non−standardized treatment regimens. Although some studies have reported the application of molecular classification in EAH patients, the evidence remains limited. These confounding factors may hinder an accurate assessment of treatment efficacy in patients with endometrial cancer. Nevertheless, this is the first study to demonstrate the efficacy of PD−1 inhibitors in fertility−sparing treatment for patients with the dMMR subtype. Thus, the results warrant further validation in prospective, well−controlled trials. Currently, several GCP trials (e.g., NCT06914297, NCT06549855) are underway to investigate the combination of PD-1 inhibitors and progesterone in fertility−sparing treatment for patients with MMRd endometrial cancer. Further results from these studies are eagerly anticipated.

## 5 Impact of immunotherapy on fertility

In addition to achieving favorable remission and pregnancy rate, it is also important to consider whether the treatment plan affects reproduction function. Traditional fertility-sparing regimens for EC are based on progestin therapy. This regimen is the safest therapeutic option during pregnancy, whereas the immune-related safety profile of ICIs remains uncertain. Whether these agents impair female fertility remains a critical question. Current evidence on the impact of ICIs on fertility is conflicting. Some animal models show acute ovarian toxicity, a potential association with spontaneous abortion, and presumed fetal risk in humans, resulting in FDA Pregnancy Category D classification (56, 57). In contrast, preliminary clinical pharmacokinetic studies suggest no acute gonadotoxicity at clinically relevant concentrations. ICI-related toxicities most frequently affect barrier tissues-including the skin, gastrointestinal tract, liver, and respiratory epithelium-followed by endocrine organs. In anti-PD-1 therapy, pneumonitis, thyroid dysfunction, and diabetes are particularly prevalent (58).

Existing studies have identified four main categories of adverse effects: primary gonadal insufficiency, secondary gonadal insufficiency, adverse pregnancy outcomes, and changes in libido and sexual function (59, 60). More importantly, most data on pregnancy exposure to ICIs come from case reports or spontaneous reporting systems, both of which have substantial selection bias and underreporting. To date, studies on the effects of immunotherapy on fertility, pregnancy, and offspring remain limited, and high-level evidence to guide clinical decisions is lacking.

### 5.1 Ovarian function

Regarding ovarian tissue, PD-1 inhibitors may increase T-cell infiltration into the ovary, disrupting immune homeostasis in the follicular microenvironment and impairing follicular development. Conversely, these inhibitors may overactivate inflammatory cascades (e.g., via cytokines such as IL-6 and TNF-α), leading to ovarian dysfunction. However, studies invesgating the impact of immunotherapy on ovarian function remain limited and show inconsistent results. Winship et al. evaluated PD-L1 and CTLA-4 inhibitor effects on the ovary in tomor-bearing and tumor-free mouse models. ICIs increased intraovarian immune cell infiltration and TNF-α expression, reduced ovarian follicle reserve, and impaired oocyte maturation and ovulation (61). In contrast, to assess the impact of PD-1 inhibition on ovarian function, Zheng et al. combined Mendelian randomization (MR) with experimental validation. Their results showed that PD-1 inhibitors at clinically relevant concentrations do not induce acute ovarian toxicity (62). Although long-term effects require further study, these findings suggest that standard anti-PD-1 immunotherapy does not impair follicle survival or endocrine function.

### 5.2 Endocrine toxicity

Approximately 40% of patients receiving ICI therapy experience immune-related endocrine adverse events, including hypophysitis, thyroid dysfunction, adrenalitis, and autoimmune diabetes. These events typically arise within six months of treatment initiation (63).

Thyroid dysfunction is the most common ICI-related endocrinopathy, occurring in approximately 10% of patients on anti-PD-1/PD-L1 monotherapy, with hypothyroidism representing the majority of cases. Hypothyroidism impairs fertility through menstrual irregularities, disruption of follicular development and ovulation, reduced conception rates, and increased risks of miscarriage and preterm birth (64).

Hypophysitis is relatively rare with anti-PD-1/PD-L1 monotherapy (incidence: 0.5-1%). Fertility−relevant manifestations include secondary adrenal insufficiency, secondary hypogonadism, and secondary hypothyroidism. Secondary adrenal insufficiency can disrupt female sex hormone secretion, impairing menstrual cycles, ovulation, and endometrial receptivity, thus increasing infertility risk (65, 66).

ICI−related diabetes mellitus (ICI−DM) has an incidence slightly below 1%. Approximately 97% of reported ICI−DM cases occur in patients on anti−PD−1/PD−L1 therapy or combination regimens. Diabetes impairs female fertility primarily through ovarian dysfunction-including ovulatory disorders and luteal phase insufficiency-and also increases pregnancy complication risks (e.g., miscarriage, preterm birth, fetal malformations) while reducing endometrial receptivity (67, 68).

### 5.3 Pregnancy outcomes

Physiologically, the PD−1/PD−L1 pathway is essential for maintaining maternal immune tolerance to the developing fetus. As pregnancy progresses, PD−L1 expression on T cells at the maternal–fetal interface increases to prevent *in utero* fetal rejection. Blockade of these pathways could theoretically trigger fetal−directed immune responses. Moreover, ICI therapy may exert negative effects on maternal–fetal immune tolerance even months or years after treatment cessation (60). Guleria et al. reported that PD−L2 is expressed throughout the decidua, whereas PD−L1 expression is restricted to the basal decidua. Studies have confirmed that PD−L1 is a key negative regulator of the maternal allogeneic immune response to fetal antigens *in vivo (*56). Noseda et al. described 103 safety reports of perinatal ICI exposure using the WHO spontaneous reporting system VigiBase^®^. Among 56 reports with pregnancy-related outcomes, adverse events included spontaneous abortion, fetal growth restriction, and preterm birth (60). Although animal models exposed to anti-PD-L1 agents showed an elevated miscarriage risk, congenital malformation rates in surviving infants did not appear increased (57, 69, 70).

Regarding fetal and neonatal outcomes, a limited number of safety reports documented successful pregnancies without a consistent pattern of major anomalies or characteristic immune-related events. Le-Nguyen et al. reported a successful pregnancy and healthy neonatal outcome in a patient with Hodgkin lymphoma who received pembrolizumab. After achieving durable complete remission, the patient conceived two years following treatment discontinuation and delivered a healthy boy (71). Wen et al. reported a successful pregnancy in a patient with metastatic melanoma receiving combined CTLA-4 and anti-PD1 therapy. The patient was found to be at seven weeks of gestation during maintenance nivolumab monotherapy after 14 months of treatment; therapy was discontinued once pregnancy was diagnosed. She experienced threatened preterm birth at 33 weeks and underwent cesarean delivery. The infant presented with severe intrauterine growth restriction and congenital hypothyroidism. At six months postpartum, the patient maintained a complete response, and the infant’s weight gain followed the standard growth curve (72).

### 5.4 Contraception

Pregnancy during ICI therapy carries risks of fetal loss, preterm birth, and growth restriction based on animal and limited human data, and ICIs may impair ovarian reserve in animal models, although human evidence remains inconclusive (54, 60, 73). Therefore, the NCCN guidelines recommend that patients of childbearing age use effective contraception during immunotherapy and for at least five months after treatment completion. This period corresponds to at least five to ten half−lives, depending on the specific agent. Notably, receptor occupancy at the individual level may vary due to multiple factors, including tumor burden and genetic polymorphisms that affect neonatal Fc receptors. This variability suggests that these antibodies may have prolonged functional effects on their targets, with unknown consequences should conception or pregnancy occur during this window. Most clinical trials also require that women of reproductive age use at least two forms of contraception for six months after anti-PD-1/PD-L1 therapy. Although research on the effects of ICIs on fertility remains limited, clinical practice should prioritize reproductive risk mitigation. Strategies include early referral for fertility evaluation and gamete cryopreservation before initiating immunotherapy (74, 75).

## 6 Ethics

According to the 2023 ESGO/ESHRE/ESGE joint guidelines, the ethical core of FST for endometrial cancer lies in balancing patients’ reproductive autonomy with oncologic safety. The guideline emphasizes that FST is not a standard approach and carries recurrence risks-especially for Lynch syndrome patients. Therefore, full informed consent and multidisciplinary team (MDT)-based shared decision-making are ethical prerequisites. Specialist referral and risk counseling are explicitly recommended by the guideline for genetically at-risk patients. Moreover, repeated FST attempts in the setting of disease recurrence require rigorous, case-by-case ethical and clinical evaluation (12). Pregnancy exposure cases should be reported to pharmacovigilance systems and enrolled in pregnancy registries to accumulate evidence, with long-term health follow-up of exposed children being essential (60, 73).

## 7 Discussion

Based on the literature reviewed in this article, current researches on molecular classification−guided FST and the application of ICIs in endometrial cancer share common methodological limitations that warrant cautious interpretation of conclusions:

### 7.1 Low-level study design

Most available evidence is derived from retrospective case series or cohort studies rather than prospective, multi-center randomized controlled trials. This introduces inherent selection bias and inadequate control of confounding factors (as described in Sections 2 and 4), making definitive causal relationships difficult to establish.

### 7.2 Insufficient sample size and high heterogeneity

The total number of FST patients is limited. Stratification by molecular classification (e.g., POLE−mut, P53abn) yields very small sample sizes, precluding stable effect estimates for clinical decision−making. Variations in inclusion criteria (e.g., inclusion of atypical hyperplasia), treatment regimens (progestin type, dose, and route), and outcome definitions (timing of CR assessment) undermine cross−study comparability and meta−analysis reliability, as reflected by the conflicting findings on molecular classification’s predictive value in Section 3.

### 7.3 Incomplete reporting of key outcomes

Oncologic outcomes such as complete remission and recurrence are well reported, but reproductive outcomes-particularly pregnancy and live birth rates stratified by fertility desire-and long−term fertility impacts (e.g., ovarian reserve markers) remains inadequate or non−standardized, creating critical evidence gaps regarding ICI−related reproductive toxicity, as noted in Section 5.

Therefore, rigorously designed prospective studies with uniform clinical outcome reporting are urgently needed to generate higher−quality evidence to inform clinical practice.

## 8 Conclusions

**Figure 2:**
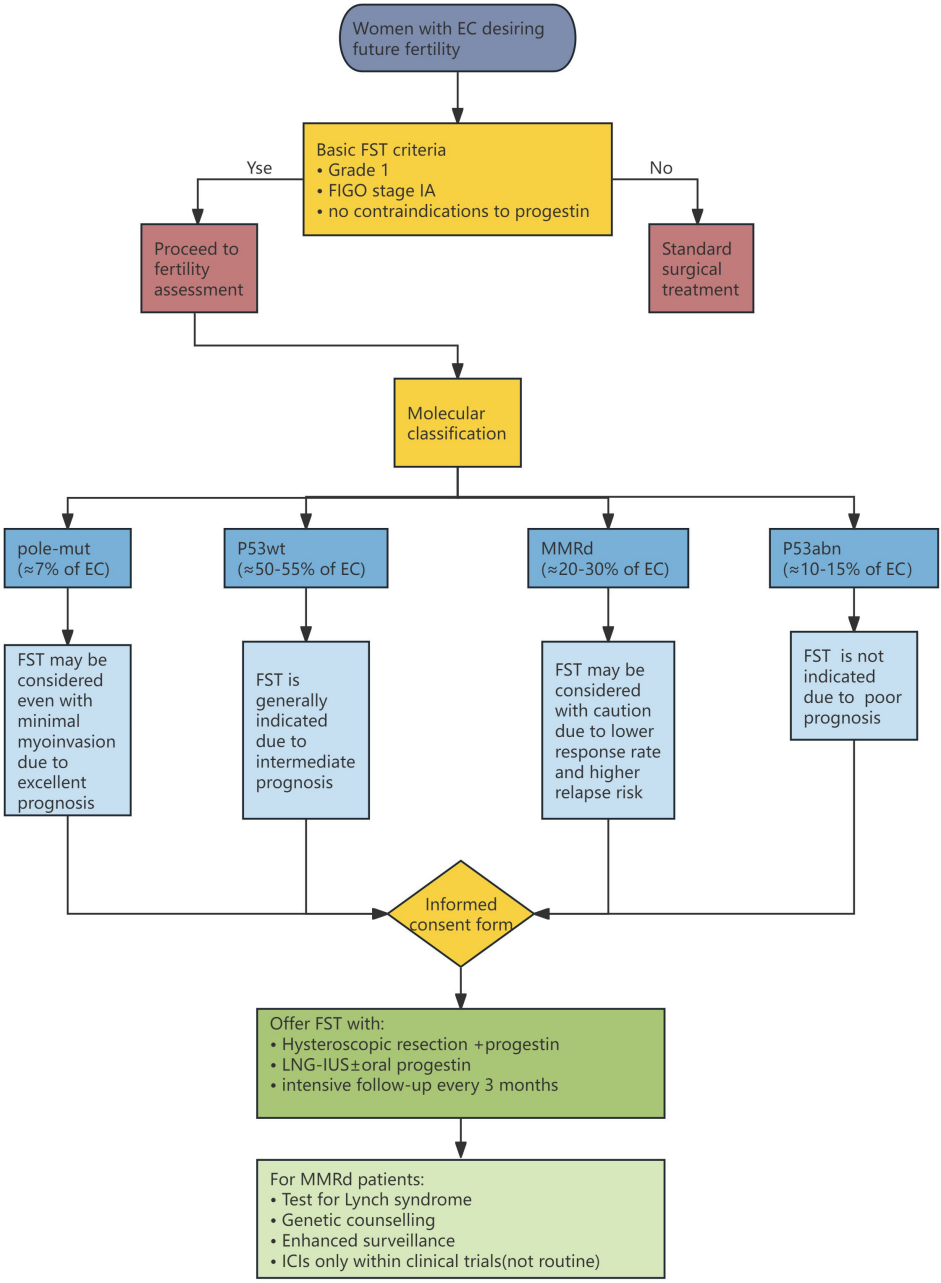
Proposed algorithm for FST in young women with endometrial cancer based on molecular classification. Molecular classification follows the ProMisE system. POLE-mut tumours have an excellent prognosis; FST may be considered even with minimal myometrial invasion (investigational). P53-wt tumours are suitable for standard FST. MMRd tumours are associated with lower response and higher relapse rates; FST may be offered with caution, mandatory Lynch syndrome testing, and enhanced surveillance. ICIs are not routinely recommended for FST outside clinical trials due to insufficient reproductive safety data. P53abn tumours have a poor prognosis and are not suitable for any FST; hysterectomy is indicated.

Although molecular classification has been widely adopted in endometrial cancer, its role in fertility−sparing treatment (FST) remains to be fully defined, and current evidence does not support its use as an independent criterion for clinical decision−making. Available studies indicate that patients with dMMR tumors have lower complete remission rates and higher recurrence risks with conventional progestin−based FST compared with other subtypes. Given its extremely poor prognosis, the p53abn subtype is generally ineligible for any fertility−sparing therapy. In contrast, POLE−mut tumors, despite often being high−grade, have an excellent prognosis that provides a theoretical rationale for expanding conservative treatment indications. Nevertheless, these conclusions are mostly drawn from small, retrospective studies with high heterogeneity, and molecular classification should be viewed as clinical adjunctive information rather than definitive evidence. For patients undergoing FST, thorough fertility evaluation, strict adherence to inclusion criteria, and intensive follow−up are essential. Given the heterogeneity of oncologic outcomes across molecular subtypes, a practical algorithm incorporating these data into decision−making is warranted, as presented in **Figure 2**.

For special populations—including patients with grade 2 tumors, minimal myometrial invasion (1–2 mm), or Lynch syndrome—fertility−sparing treatment using immune checkpoint inhibitors (ICIs) should be considered with particular caution. Although the ESMO guidelines classify endometrial cancer into low−grade (grade 1 and 2) and high−grade (grade 3) categories, the safety and efficacy of ICIs in these special subgroups remain unproven. Therefore, when immunotherapy is considered for FST in these patients, rigorous follow−up with frequent endometrial assessment (every 3–6 months) and multidisciplinary team (MDT) management is mandatory to ensure timely detection of disease progression or recurrence.

Given the established efficacy of ICIs in recurrent or advanced endometrial cancer, their potential role in FST has been investigated. Nevertheless, the use of ICIs in this setting remains investigational and is not recommended for routine clinical practice, with several GCP−compliant trials currently ongoing. Further prospective studies are urgently needed to determine whether ICIs adversely affect fertility, pregnancy, and offspring outcomes, and to establish evidence−based recommendations for these challenging scenarios.
